# Season of Birth and Dopamine Receptor Gene Associations with Impulsivity, Sensation Seeking and Reproductive Behaviors

**DOI:** 10.1371/journal.pone.0001216

**Published:** 2007-11-21

**Authors:** Dan T. A. Eisenberg, Benjamin Campbell, James MacKillop, J. Koji Lum, David S. Wilson

**Affiliations:** 1 Department of Anthropology, Northwestern University, Evanston, Illinois, United States of America; 2 Department of Anthropology, State University of New York at Binghamton, Binghamton, New York, United States of America; 3 Department of Biology, State University of New York at Binghamton, Binghamton, New York, United States of America; 4 Laboratory of Evolutionary Anthropology and Health, State University of New York at Binghamton, Binghamton, New York, United States of America; 5 Department of Anthropology, University of Wisconsin Milwaukee, Milwaukee, Wisconsin, United States of America; 6 Department of Psychology, State University of New York at Binghamton, Binghamton, New York, United States of America; 7 Center for Alcohol and Addiction Studies, Brown University, Providence, Rhode Island, United States of America; University of Maryland, United States of America

## Abstract

**Background:**

Season of birth (SOB) has been associated with many physiological and psychological traits including novelty seeking and sensation seeking. Similar traits have been associated with genetic polymorphisms in the dopamine system. SOB and dopamine receptor genetic polymorphisms may independently and interactively influence similar behaviors through their common effects on the dopaminergic system.

**Methodology/Principal Findings:**

Based on a sample of 195 subjects, we examined whether SOB was associated with impulsivity, sensation seeking and reproductive behaviors. Additionally we examined potential interactions of dopamine receptor genes with SOB for the same set of traits. Phenotypes were evaluated using the Sociosexual Orientation Inventory, the Barratt Impulsivity Scale, the Eysenck Impulsivity Questionnaire, the Sensation Seeking Scale, and the Delay Discounting Task. Subjects were also asked about their age at first sex as well as their desired age at the birth of their first child. The dopamine gene polymorphisms examined were Dopamine Receptor D2 (*DRD2*) *TaqI A* and D4 (*DRD4*) *48 bp VNTR*. Primary analyses included factorial gender×SOB ANOVAs or binary logistic regression models for each dependent trait. Secondary analysis extended the factorial models by also including *DRD2* and *DRD4* genotypes as independent variables. Winter-born males were more sensation seeking than non-winter born males. In factorial models including both genotype and season of birth as variables, two previously unobserved effects were discovered: (1) a SOB×*DRD4* interaction effect on venturesomeness and (2) a DRD2×*DRD4* interaction effect on sensation seeking.

**Conclusion:**

These results are consistent with past findings that SOB is related to sensation seeking. Additionally, these results provide tentative support for the hypothesis that SOB modifies the behavioral expression of dopaminergic genetic polymorphism. These findings suggest that SOB should be included in future studies of risky behaviors and behavioral genetic studies of the dopamine system.

## Introduction

Season of birth (SOB) has been associated with such diverse physiological and psychological human traits as birth weight [1], adult height [2], [3], body-mass index (BMI = weight in kg/height in m^2^) [4], [5], eating disorders [6], blood pressure [7], life expectancy [8], handedness [9], age of menarche [10], fecundability [11], sex-ratio of offspring [12], age at menopause [10], suicide[13]–[16], schizophrenia [17], [18], autism [19], panic disorder [20], university grades [21] and morning versus evening preference [22]–[24]. Of particular interest for our study, winter-borns exhibit increased novelty seeking [25]–[27] and sensation seeking [28] relative to those born during the remainder of the year.

Suggested explanations for the associations between SOB and this wide array of psychological and physiological phenotypes include variations in infectious disease exposure, nutrition, temperature, maternal hormones, maternal egg quality, birth complications and photoperiod [6], [17], [29]. Photoperiod is perhaps the best explored and supported hypothesis as to why SOB bears an association with risky behaviors. It is hypothesized that variation in daylight during gestation or perinatally impacts the dopamine-melatonin balance regulating circadian and seasonal rhythms and serotonin turnover [24], [28], [30], [31]. The serotonin metabolite, 5-hydroxyindoleacetic acid (5-HIAA) and dopamine metabolite, homovanillic acid (HVA) have both been shown to vary with SOB [31]. Consistent with an effect of photoperiod on risky-behaviors, HVA increases with increasing novelty seeking and dopamine turnover reaches its extremes with the solstices [30]. A somewhat different type of risky behavior, suicide, also seems to be inter-related with SOB and 5-HIAA [30].

Consistent with the dopamine-melatonin hypothesis, traits associated with SOB have also been linked to genetic polymorphisms in the dopamine system. These include BMI [32]–[34], eating disorders [35]–[37], blood pressure [38], fertility [39]–[41], novelty seeking [42], [43], [but 44], and sensation seeking [45]. Additionally, there is evidence of interactions between SOB and specific genetic polymorphisms, such as the dopamine receptor D4 (*DRD4*) *48 bp VNTR* polymorphism, on psychiatric disorders [46], [47] and BMI [5]. In fact, SOB effects may be directly related to *DRD4*. In the retina DRD4 mRNA has been found in photoreceptor cells that indirectly control melatonin synthesis and have a regulatory role on light sensitive cyclic adenosine monophosphate [48]–[51]. Additionally, dopamine has been found to inhibit retinal melatonin synthesis via D2/D4 receptors but not through D1/D5 receptors [52].

This paper focuses on SOB and its interaction with dopamine receptor genes and gender. It is part of a larger series of studies examining the associations of genetic polymorphisms of the dopamine system with behavior [53], [54]. In this case, we examined how SOB was associated with measures of sensation seeking, impulsivity and reproductive behaviors, as well as the interaction between SOB, gender, DRD2 and DRD4 polymorphisms in influencing these behavioral outcomes. The phenotypes were evaluated using the Sociosexual Orientation Inventory (SOI), Barratt Impulsivity Scale (BIS), Eysenck Impulsivity Questionnaire (EIQ), Sensation Seeking Scale (SSS), and Delay Discounting Task (DDT). Additional phenotypes considered included self-reported virginity status, age at first sexual intercourse, and desired age at first birth. Three independent scales (BIS, EIQ, and DDT) were used to assess impulsivity because of the heterogeneous nature of impulsivity [reviewed in 54]. This is the first such study we are aware of to examine the interactions between SOB and dopamine genetic polymorphisms on normal behavioral variation in a non-clinical population.

We examined two dopaminergic genetic polymorphisms: *DRD2 TaqI A* and *DRD4 48 bp VNTR*. The *DRD2 TaqI A* site is a single nucleotide polymorphism (SNP) with a major A2 allele, and minor A1 allele. The A1+ genotype (heterozygous or homozygous A1) has been most strongly associated with substance abuse, particularly alcoholism, albeit with some controversy [55]. The A1+ genotype has also been related to pathological gambling, novelty seeking, and sensation seeking [45], [55]. The *DRD2 TaqI A* site is 9.4 kb downstream from the coding region for the dopamine D2 receptor gene. It is not in any known regulatory region, and although the A1 allele is associated with a decrease in dopamine D2 binding and glucose metabolic rates in many brain regions [55]–[57], the mechanism by which it affects *DRD2* expression is unknown. The *TaqI A* polymorphism is also in a nearby kinase gene, the *Ankyrin Repeat and Kinase Domain Containing 1 (ANKK1)* gene, where it causes a Glutamate→Lysine substitution [58], [59]. The results of the amino acid substitution are not known, but could impact interactions of the ANKK1 protein with other proteins including the dopamine D2 receptor [59]. No other polymorphism has been revealed in linkage disequilibrium with *TaqI* A that could easily account for these associations [41], [58]–[60].

The *DRD4 48-bp VNTR* polymorphism is in exon 3 of the gene coding for the dopamine receptor D4. The *VNTR* polymorphism varies between 2 and 11 repeats of a similar 48-bp coding region sequence, with a trimodal distribution of 2, 4 and 7 repeat alleles (2R, 4R and 7R) in most, but not all, populations [61]. Although the functional significance of the *DRD4 VNTR* polymorphism has not been definitively characterized, long alleles (typically 7R as opposed to 4R) have been generally found to be functionally less reactive in in-vitro expression experiments [62]–[66], with some heterogeneity [67]–[71]. Additionally, in vivo human pharmacological studies are also generally consistent with the notion that 7R alleles are associated with less responsive D4 receptors than 4R alleles [72]–[76].

Based on the existing literature, we predicted that winter-borns would exhibit increased sensation seeking, increased impulsivity, more promiscuous sexual behavior and a desire for children earlier. We further predicted that being winter-born would potentiate the effects of risk-conferring dopamine receptor alleles on these behavioral traits.

## Methods

### Participants and procedures

Between February and April 2005 a total of 195 subjects were recruited for participation from the Human Subject Research Pool at the State University of New York at Binghamton, U.S.A. The subject pool draws on mostly full time students currently in psychology courses who participate in research studies for course credit. No screening measures were placed on who could participate. All procedures were approved by the Human Subjects Research Review Committee at the State University of New York at Binghamton and all subjects gave informed consent. Participants attended group sessions (maximum = 10), where they were first provided with oral instructions followed by DNA sample collection. Participants then completed the delay discounting task followed by the self-report measures administered in random order. In addition to the oral instructions, the DDT task and other measures were accompanied by on-screen written instructions and experimenters were available throughout the sessions for questions. The sessions lasted approximately one hour. All data were collected via personal computers.

### Phenotype Assessment

#### Sociosexual Orientation Inventory (SOI)

The SOI measures restriction of sexual and pair-bonding behaviors. It has been validated on student populations around the world [77], [78]. Those who score lower on the SOI generally engage in sex later in relationships that are ‘characterized by reliably greater expressed love, dependency, commitment and investment’ [77, p. 876]. The SOI was slightly altered to differentiate heterosexual and homosexual activity and sexual identity, but because the sample contained only one subject self-identified as bisexual and four self-identified as homosexual, they were not distinguished from those self-identified as heterosexual in the further analyses.

#### Barratt Impulsivity Scale, Version 11 (BIS-11)

The BIS-11 provides a general measure of impulsivity and three subscores: Attentional Impulsiveness (BIS-AI), Motor Impulsiveness (BIS-MI) and Non-Planning Impulsiveness (BIS-NPI). The BIS-11 has undergone psychometric validation [79].

#### Eysenck Impulsivity Questionnaire (EIQ)

The EIQ is a self-report measure of impulsivity that generates three subscales, of which two were relevant to the current study: Impulsiveness (EIQ-I), and Venturesomeness [EIQ–V; 80]. The EIQ has undergone psychometric validation [e.g., 81].

#### Sensation Seeking Scale–Form A (SSS)

Sensation seeking is a related construct to impulsivity, and has both been shown to exhibit moderate positive correlations with self-reported impulsivity and to potentially share genetically-mediated common biological mechanisms with impulsivity [82]. The SSS is a psychometrically validated measure [83] that provides an overall measure of sensation seeking proneness (SSS-Total) and four relevant lower order factors: Experience Seeking (SSS-ES), Boredom Susceptibility (SSS-BS), Disinhibition (SSS-D), and Thrill and Adventure Seeking (SSS-TAS).

#### *Delay Discounting Task* (DDT)

To capture discounting of delayed rewards empirically, the DDT poses participants with repeated choices between a smaller reward received immediately and a greater reward received after some time delay (e.g., “Would you prefer to have $65 today or $100 in a month?”). Over the course of the task, the amounts of immediate rewards are successively modified, as is the duration of delay. The individual's responses to the entire array of choices are then used to empirically derive their discounting function (i.e., how steeply they discount delayed rewards relative to immediate rewards, commonly denoted *k*). The DDT was administered with hypothetical money via a custom computer program [84] which is fully described in Supplementary File S1. Model fits of how well subjects' discounting functions fit Mazur's [85] nonlinear equation used to derive *k* values are calculated as *R^2^* values. Erratic subjects and those with *R^2^* values below 0.30 were excluded from principal analyses [86]. The DDT *k* value was normalized with a logarithmic transformation, as is typical in delay discounting research.

#### Additional Self-Report Questions

Subjects were asked their desired age to have their first child, or whether they did not want to have children. They were also asked their age at first sex, or if they were virgins.

#### Season of birth

Subjects were asked their year, month and country of birth. For SOB analyses, only those born under 25 years ago in countries that are predominately north of the Tropic of Cancer were considered. North of the Tropic of Cancer marks increased photoperiod variation in a consistent direction and a point beyond which photoperiod variation has been observed to effect reproductive behavior in non-human primates [87]. Since most of the sample (181 or 92.8%) was born north of the Tropic of Cancer, analysis was only conducted on this Northern Hemisphere sample. The 25 year age limit was employed because SOB effects have been observed to reverse with age in other studies [24], [26]–[28] and all but two subjects were under 25 in this study.

Consistent with past associations of SOB with sensation and novelty seeking [26]–[28], October to March borns are classified as high risk-conferring winter-borns relative to those born in the remainder of the year (not-winter-borns). Because other methods of parsing SOB have been employed in the literature, we include our raw dataset as Supplementary File S2 to allow further analysis by researchers who may want to examine hypotheses beyond the ones we consider here.

### Genotyping

DNA was collected with QuickExtract buccal swabs and extracted with BuccalAmp solution as directed by the manufacturer (Epicenter). Subjects were instructed to rinse their mouths out with water before swabbing. *DRD2 TaqI A* was typed with a PCR/RFLP method [based on 88] described completely in Supplementary File S3. The *DRD4 VNTR* locus was genotyped using an adaptation of a previous protocol [89] described fully in Supplementary File 3. Allele frequency data was submitted to The Allele Frequency Database (ALFRED; http://alfred.med.yale.edu/alfred/sampleDescrip.aspsampleID001775)').

### Data Analysis

All data were examined for outlying data points, distribution normality, and missing values. To assure missing responses were not systematically biased by SOB, missing versus non-missing data for each phenotype scale was analyzed by SOB in 2×2 contingency tables. Examination of bias in missing data by *DRD2 TaqI A* and *DRD4 VNTR* genotypes has been conducted previously and are not reported on here [53], [54]. Missing values were not imputed, but excluded from analysis. Fits to HW equilibria were tested with the HWE program [90], with which the *DRD2* HW equilibrium was tested with Fisher's Exact and *DRD4* with the Markov Chain algorithm.

Based on previous association studies of the *DRD2 TaqI A* polymorphism, individuals with at least one A1 allele were designated as A1+ and those who were homozygous for the A2 allele were designated A1-. Similarly, *DRD4 48 bp VNTR* genotypes were separated into long allele (7 repeats or longer) present (L+) and long allele absent (L−) groups. The principal data analyses used a tiered analysis. First, since several past studies have found that SOB effects vary by gender, factorial 2 (Male/Female)×2 (Winter-Born/Not-Winter-Born) ANOVAs were conducted. Then to see if genotype interacted with SOB to effect the traits in question, *DRD2* and *DRD4* genotypes were added to each factorial model (2 [Male/Female]×2 [Winter-Born/No-Winter-Born]×2 [A1+/A1−]×2 [L+/L−], although four-way interactions were considered uninterpretable and are not discussed). Similarly, categorical dependent variables were first analyzed with forward conditional factorial binary logistic regression models with dichotomized SOB and gender and then secondarily include *DRD2* and *DRD4*. Direct SOB effects and SOB interactions with genotypes are the main focus of the study. Since this study is only concerned with sexual dimorphism in so far as it moderates SOB or genotype associations, main effects of gender are not commented on. All scales used in this study were previously examined in relation to the genetic polymorphisms and gender [53], [54] and these results are not reported on here unless the inclusion of SOB in statistical models reveals new results.

Since this study employs multiple phenotypic scales, the potential exists for type I errors. For several reasons we have not employed a correction of our significance criteria for multiple testing [91], [92]. The diversity of scales employed in this study should not decrease the sensitivity of analysis. In addition, because of the exploratory nature of our analysis [91], and because the phenotypic variables are often correlated (see Table 1 and Results and Discussion), corrections for multiple tests are too conservative [92]. Nonetheless, to address the risk of Type I errors, we have reduced our significance criteria from the traditional≤.05 to a≤.01 level for the principal analyses (for the correlation matrix in Table 1 a traditional≤.05 criteria is employed). It is important for the reader to bear these cautions in mind as they interpret the results.

**Table 1:** Correlation among continuous behavioral phenotype scales.

	AgeSex	Kids	SOI	DDT	BIS-AI	BIS-MI	BIS-NPI	BIS-Total	EIQ-V	EIQ-I	SSS-D	SSS-ES	SSS-BS	SSS-TAS
AgeSex	1.00													
Kids	0.09	1.00												
SOI	−0.21*	0.09	1.00											
DDT	0.07	−0.06	0.16	1.00										
BIS-AI	−0.09	−0.06	0.30**	0.08	1.00									
BIS-MI	−0.02	0.10	0.33**	0.13	0.54**	1.00								
BIS-NPI	0.01	0.03	0.16	0.12	0.45*	0.49**	1.00							
BIS-Total	−0.04	0.02	0.32**	0.15	0.80**	0.84**	0.81**	1.00						
EIQ-V	−0.07	0.13	0.19**	0.01	0.11	0.24**	0.11	0.19*	1.00					
EIQ-I	−0.01	−0.01	0.41**	0.21*	0.53**	0.56**	0.44**	0.63**	0.25**	1.00				
SSS-D	−0.19*	0.20*	0.55**	0.12	0.30**	0.34**	0.23**	0.35**	0.24**	0.42**	1.00			
SSS-ES	−0.06	−0.03	0.19**	−0.01	0.12	0.20**	0.03	0.12	0.46**	0.23**	0.19*	1.00		
SSS-BS	−0.07	0.09	0.47**	0.07	0.31**	0.36**	0.28**	0.39**	0.30*	0.51**	0.44**	0.28**	1.00	
SSS-TAS	0.06	0.11	0.02	−0.04	0.02	0.19**	0.06	0.11	0.80**	0.05	0.15*	0.37**	0.10	1.00
SSS-Total	−0.06	0.15	0.47**	0.04	0.28**	0.41**	0.23**	0.38**	0.69**	0.44**	0.68**	0.65**	0.64**	0.66**
*≤**.05**	**≤**.01**													

Table Variable definitions given in Table 2.

## Results

### Sample Background

The sample is described demographically in Table 2. It had a roughly equal sex ratio, a narrow age range (as expected in a college population) and was predominately of European descent. Genotype and allele frequencies are given for *DRD2 TaqI A* and *DRD4 48-bp VNTR* in Supplementary File S3. Genotype frequencies were comparable to other samples of mixed populations with predominately European descent. Both loci were in Hardy-Weinberg Equilibrium (*DRD2,* Fisher's Exact test *p* = 1.0; *DRD4 VNTR,* Markov Chain Algorithm *p* = 0.38). Descriptive statistics of each dependent variable are given in Table 3. As can be seen in Table 1, where the correlations between continuous dependent variables are given, with the exception of AgeSex (age at first sex), Kids (desired age at first child) and DDT, the correlations between scales are all positive, frequently significant, and generally high. Correlations among each gender separated can be found in Supplementary File S4.

**Table 2:** Demographic Information

Variable	Descriptive Statistics
Sex	42% male; 58% female
Age	Median = 19.33 (IQR = 18.83–20.35)
Ethnicity	44.1% European, 14.4% East Asian, 11.8% Latin American, 5.1% South Asian, 3.1% Native North American, 1.5% African American, 1.0% Pacific Islander, 1.0% African, 13.8% multiracial, 5.6% unknown. (does not sum to exactly 100.0% because of rounding)

Subject Characteristics (n = 195).

**Table 3:** Descriptive Statistics of Dependent Variables

Variable	Definition	N	Mean	SD
**AgeSex**	Age at first sexual intercourse	124	16.88	1.46
**Kids**	Desired age to begin having children	163	28.91	2.56
**SOI**	Sociosexual Orientation Inventory	153	57.15	30.59
**DDT**	Delay Discounting Task (patience)	156	−1.33	0.71
**BIS**	Barratt Impulsivity Scale			
** BIS-AI**	Attentional Impulsiveness	169	17.21	3.46
** BIS-MI**	Motor Impulsiveness	172	21.78	3.76
** BIS-NPI**	Non-Planning Impulsiveness	166	25.46	3.97
** BIS-Total**	General Impulsiveness	157	64.43	9.21
**EIQ**	Eysenck Impulsivity Questionnaire			
** EIQ-V**	Venturesomeness	174	9.83	3.45
** EIQ-I**	Impulsiveness	168	10.48	4.34
**SSS**	Sensation Seeking Scale			
** SSS-D**	Disinhibition	169	5.89	2.43
** SSS-ES**	Experience Seeking	170	5.52	1.92
** SSS-BS**	Boredom Susceptibility	174	3.17	2.04
** SSS-TAS**	Thrill and Adventure Seeking	174	6.64	2.71
** SSS-Total**	Overall Sensation Seeking Proneness	163	21.25	5.81
**Virgins** *	Are virgins	173	28.30	
**Want Children** *	Want to have children	172	94.80	

* categorical variables: mean of percent responding positively given instead of mean

179 of the 195 subjects (91.8%) met the inclusion criteria for the SOB analysis. Winter borns made up 52.5% of the sample. SOB did not systematically vary with gender, *DRD4* or *DRD2* genotypes (not shown). Between 2.8% and 14.5% of values were missing across phenotype scales (Table 3). No heterogeneity of missing values on the phenotype scales by season of birth was found.

### Season of birth and season of birth by gender associations

**Figure 1:**
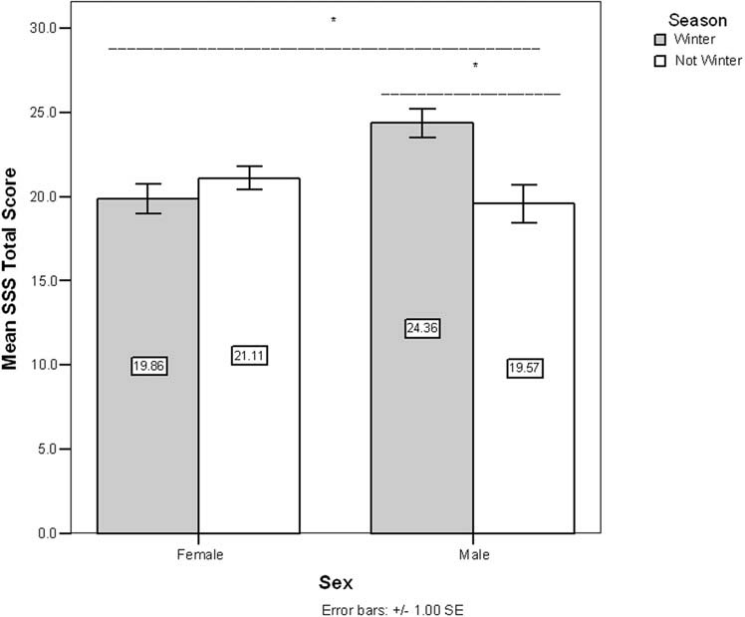
Mean Sensation Seeking proneness by Sex and SOB. SSS varied significantly by the interaction of Sex and SOB. In post-hoc analysis SSS varied by SOB in males, but not females.

Table 4 shows main effects of SOB, gender and interaction effects of SOB×gender on each phenotype. SOB×gender interactions were prominent on total sensation seeking and the sensation seeking subscores, disinhibition and boredom susceptibility. As illustrated in Figure 1 for the SSS Total score, female winter-borns were on average less sensation seeking than female not-winter-borns, while male winter borns were more sensation seeking than male not-winter borns (the same pattern was evident across the three significant SSS sub-scales). In post-hoc analysis, the differences in total sensation seeking, disinhibition and boredom susceptibility by SOB were significant in males (SSS-Total: F[1,65] = 11.520, p = .001; SSS-D: F[1,69] = 9.469, p = .003; SSS-BS: F[1,72] = 8.257, p = .005) but not females (SSS-Total: F[1,94] = 1.257, p = .265; SSS-D: F[1,96] = 1.712, p = .194; SSS-BS: F[1,98] = 1.273, p = .262). There was also a trend towards an SOB×gender interaction effect on self-reported virginity status (Table 4). This effect of SOB was similarly prominent in males, but not females (not shown).

**Table 4:** Associations of Season of Birth, Gender and Season of Birth X Gender with dependent variables.

Variable	SOB		GENDER		SOB X GENDER	
	F	P	F	P	F	P
**AgeSex**	0.73	0.394	0.55	0.460	0.459	0.500
**Kids**	0.802	0.372	5.649	0.019	2.067	0.152
**SOI**	0.485	0.487	12.524	**0.001**	0.035	0.852
**DDT**	0.425	0.515	1.841	0.177	0.066	0.798
**BIS**						
** BIS-AI**	0.088	0.767	4.13	0.044	0.005	0.944
** BIS-MI**	0.262	0.610	15.625	**<.001**	1.225	0.270
** BIS-NPI**	2.255	0.135	2.21	0.139	2.251	0.135
** BIS-Total**	0.346	0.557	9.59	**0.002**	0.54	0.464
**EIQ**						
** EIQ-V**	3.201	0.075	7.825	**0.006**	0.134	0.714
** EIQ-I**	0.008	0.929	3.136	0.078	0.542	0.462
**SSS**						
** SSS-D**	2.878	0.092	1.127	0.290	10.905	**0.001**
** SSS-ES**	0.674	0.413	0.023	0.880	1.513	0.220
** SSS-BS**	2.703	0.102	3.516	0.063	9.179	**0.003**
** SSS-TAS**	1.812	0.180	1.303	0.255	1.332	0.250
** SSS-Total**	3.936	0.049	2.767	0.098	11.457	**0.001**
**Virgins** *	0.65	0.420	0.61	0.436	5.32	0.021
**Want Children** *	0.13	0.716	0.64	0.425	0.00	0.983

* categorical variables: Wald statistic given instead of F value

Bolded values are significant (p≤.01)

### Gene by season of birth interactions-multivariate analysis

**Figure 2:**
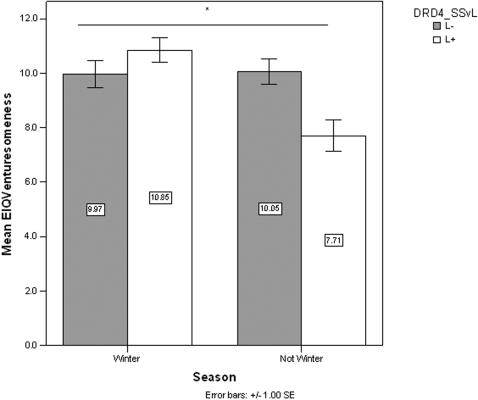
Mean EIQ Venturesomeness by DRD4 genotype and SOB. Venturesomeness varied significantly by the interaction of DRD4 and SOB.

To evaluate whether there was a moderating relationship between SOB and dopamine gene polymorphisms on the behavioral traits in this study, *DRD2* and *DRD4* were added as independent variables in the factorial ANOVA and binary logistic regression models. We found a significant interaction effect of *DRD2*×*DRD4* on total sensation seeking (SSS-Total; F [1,145] = 6.883, p = .010), which was not observed in previous models that did not include SOB. Among those without A1 alleles (A1−), long *DRD4* alleles (L+) were associated with decreased SSS-Total, but the reverse was true among those with A1 alleles (A1+). In addition, there was an interaction effect of SOB×*DRD4* on EIQ-V (F[1,156] = 9.878, p = .002) as illustrated in Figure 2. On all other scales, no new significant associations were evident.

All significant findings in the gender by SOB analysis (without genotype variables), were found in this gender×SOB×*DRD2*×*DRD4* analysis (not shown).

## Discussion

We hypothesized that winter-borns would show higher rates of risk-associated behaviors, including sensation seeking, impulsivity, and sexual promiscuity. We found support for this hypothesis only for sensation seeking and in a near-significant trend for virginity status. Additionally, we hypothesized that both winter-birth and risk-conferring alleles would together promote more risky-behaviors. We found only limited support for such an interaction. However, we did find that including SOB revealed some previously unobserved effects of the DA receptor genetic polymorphisms. On the EIQ Venturesomeness scale, the expression of a dopamine gene polymorphism (*DRD4*) seemed to be moderated by SOB.

### Season of birth

Our finding of increased sensation seeking in winter borns is generally consistent with Joinson and Nettle's finding of increased SSS in young adult winter-borns [28]. However, Joinson and Nettle found a trend of increased sensation seeking in both genders pooled, while we only found the effect in males. Our finding is also inconsistent with past studies where SOB tends to be more, not less related to novelty-seeking among females than males [25]–[27]. It is unclear why our findings diverge from those of past studies. In other studies, SOB has been shown to have opposite effects on different age groups [27], [28], suggesting cohort or development effects. Studies that found stronger effects in females were conducted on university distance learning students in the U.K. [28], general population samples in Sweden [25], [27] and a high school sample from Sweden [26]. The fact that our samples consisted of a subsection of United States (mainly full-time) college students instead of those from a general population may help account for our different findings.

It was surprising that SOB was related to sensation seeking (SSS) and potentially virginity (in 2×2 factorial models) but not other related scales such as the Eysenck Impulsivity Questionnaire (EIQ) or Barrett Impulsivity Scale (BIS) which have been associated with the dopamine system [93]–[95] and are related in this study (Table 1). However, the SSS appears to capture a distinctive element of impulsivity/risk-taking compared to other scales including the EIQ [96]. The SSS is oriented more towards participation in specific activities than the BIS or EIQ, which require subjects to implicitly compare themselves to others. Perhaps the effects of SOB on behavior are better assessed by questions more directly related to behavioral desires rather than more subjective self-conception.

While we hypothesized that SOB effects on behavior are related to dopamine-melatonin changes, with our dataset it is not possible to clearly distinguish the underlying basis for the behavioral effects of SOB. While the SSS has been related to the DA system, it has also been related to other neurotransmitters, enzymes and hormones [reviewed in 97]. However, the associations revealed when SOB and genotype were included in the same models tentatively suggests that SOB does impact the dopamine system.

### Season of birth and dopamine polymorphisms

Including SOB in genotypic models yielded two new significant findings not previously observed. One was an interaction effect between genotype and SOB. This interaction between SOB and *DRD4* in predicting venturesomeness (EIQ-V) is consistent with our hypothesis that dopamine D4 receptors may be particularly mechanistically entangled with SOB effects. Such an interaction may indicate that both SOB and DRD4 act through related dopaminergic substrates. Additionally, the DRD2 by DRD4 association revealed when SOB was included in models together with *DRD2* and *DRD4* tentatively suggest that SOB and dopamine polymorphisms control overlapping traits. This *DRD2*×*DRD4* associations with sensation seeking (SSS-Total) is consistent with past findings of an interaction between *DRD2* and *DRD4* on impulsivity, where similarly the most extreme impulsive individuals were A1+/L+ [54].

The results presented here provide limited support for the hypothesis that SOB modulates expression of genetic polymorphisms of the dopamine system. Thus they add to previously published studies that have documented SOB interactions with the expression of dopamine system genetic polymorphisms, including interactive effects on psychiatric disorders [46], [47] and BMI in women with seasonal affective disorder [5]. However, given the limited sample size as well as the factorial nature of our statistical models, and the possibility of type I error, the results must be considered tentative.

### Evolution of Season of Birth Influences on Behavior

Circadian rhythms have been found in virtually all organisms that have been studied [98] and the coupling of photoperiod and neuroendocrine control of reproductive physiology and behavior is often important among mammals. A mammalian species complex that spans wide latidunal swaths shows clinal variation in its responsiveness to photoperiod (from no response at all to a high responsiveness), suggesting rapid evolution [87]. In several non-human primates that live in the higher latitudes of the tropics and lower temperate zone, photoperiod has been associated with reproductive and behavioral changes [87].

This rapid evolutionary potential may also be innate in humans, and subject to natural selection. Although not readily apparent, SOB could be correlated with other factors that serve as reliable cues of the environmental quality an individual is likely to experience throughout their lifetime (e.g. infectious disease load or nutrition). In order for birth environment to serve as an adaptive cue to change behaviors, birth environment must be reliably correlated to the environment experienced many years later. Alternatively, SOB may cause adaptive behaviors in infants that are selectively advantageous enough to outweigh maladaptive behaviors later in life. While it is difficult to imagine how birth climate, would be a reliable signal of future environments beyond perhaps a few years [99], [100], studies of the paleo-climate may yield another answer.

One possible way such signals could be functional is if climate changes were a persistent survival problem for humans. There is evidence that “during the present (Holocene) interglacial…cold and dry phases…[occurred]… on a 1500-year cycle, and with climate transitions on a decade-to-century timescale” [101]. On a smaller timescale, over the last millennium of Chinese history, climate changes to cold phases have been associated with decreased harvests, increased warfare, decreasing population and dynastic changes [102]. While very speculative, it is possible that physiological and behavioral plasticity based on birth environment allows better survival through such turbulent changes. Among early humans living predominantly in a tropical environment such a signal for plasticity may not have been obscured by the more marked seasonal variations now experienced farther from the equator.

Alternatively, early photoperiod may have non-adaptive effects on development. Stressed pregnant rats have offspring with altered physiologies and behavior including altered dopamine levels [103]–[106]. Early rearing environment of rhesus monkeys is associated with lower amine activity, including that of HVA [107]. Studies in rats and mice show that changes in light exposure early in life is related to sensitivity to light later in life [reviewed in 23]. These studies give reason to believe that developing human brains may be easily affected by early photoperiod. In such a long-lived species, these effects could represent compensation for early perturbations in development, rather than adaptive tracks for later-life behaviors based on early photoperiod.

### Summary

Our results tentatively suggest that SOB has a different, but related, psychological impact than dopamine D2/D4 receptor genetic polymorphisms on several behavioral phenotypes. This study replicates the past findings that winter-borns are more sensation seeking, but differs in that the association was only evident in males. SOB was unrelated to several behavioral/psychological phenotypes that were associated with *DRD2* and *DRD4* genetic polymorphisms. But, including SOB in factorial models revealed a previously unobserved association and evidence of moderation of *DRD4* expression by SOB. These results must be viewed with caution as the number of phenotypic dependent variables analyzed here may have increased the risk of type I errors. An adaptive basis for the associations found here and in other studies of SOB is not clear. The behavioral implications of SOB remain ambiguous and its interactions with genotype effects are tentative. To further dissect the association of season of birth with later behaviors, more comprehensive analysis including experimental variations of photoperiod in lab animals in utero and early in life, and analysis of light exposure of pregnant women and later their young children should be conducted. However, SOB is an easy variable to collect (requiring only knowledge of the date and location of birth of subjects) that may help elucidate behavioral genetic associations. The current results along with those of past studies provide ample reason to include season of birth as at least a control in future studies of impulsivity/risky and sensation-seeking behaviors. Furthermore, season of birth should be included in future behavior genetic studies of the dopamine system.

## Supporting Information

File S1 Delay Discounting Protocol
(0.04 MB DOC)

File S2 Datafile. Complete dataset from the study.
(0.15 MB XLS)

File S3 Genotype Protocols and Genotype and Allele Frequencies
(0.07 MB DOC)

File S4 Correlations Between Continuous Variables. Use Tabs to toggle between Female, Male and Combined Male and Female Correlations.
(0.05 MB XLS)
