# Supplementary material for: Season of Birth and Dopamine Receptor Gene Associations with Impulsivity, Sensation Seeking and Reproductive Behaviors
Source: PLoS One. 2007 Nov 21;2(11):e1216. doi: 10.1371/journal.pone.0001216 (PMC2075470; doi:10.1371/journal.pone.0001216)
Supplement: File S1 — Delay Discounting Protocol (0.04 MB DOC) [file pone.0001216.s001.doc]

**Delay Discounting Protocol**

The delayed reward was a $100 hypothetical reward to be received in the future compared to 29 reward amounts to be received today; the amounts were as follows: $100, $99.90, $99.50, $99, $96, $92, $85, $80, $75, $70, $65, $60, $55, $50, $45, $40, $35, $30, $25, $20, $15, $10, $8, $6, $4, $2, $1, $0.50, $0.10. Delay time intervals were: 1 week, 2 weeks, 2 months, 6 months, 1 year, 5 years and 25 years. These parameters were based on a previous version of the task [1, 2].

Procedurally, two reciprocal assessments were made. The first used successively decreasing amounts of immediate reward to identify the participant’s point at which they first opted to switch from selecting the discounted reward (e.g., $90) to the delayed full reward ($100) for each increment of time. After the participant switched from choosing the immediate reward, the procedure continued for five additional monetary increments after the participant switched choices to ensure this was indeed the lowest choice. After determining the descending switching point for all seven time intervals, the procedure began again at the first time interval and, starting at the increment five units below the previously determined switching point, successively increased the immediate rewards available until the individual stopped selecting the delayed reward ($100) and returned to accepting the immediate reward. Similarly, the procedure continued for five additional reward levels beyond the switch in decision-making to ensure this point was correctly identified.

To quantify participants’ discounting functions of $100 based on its delay in time, the descending and ascending switching points were averaged for each time interval, creating the point of indifference (POIs) for that interval. The POIs were then employed in the following equation to derive the function used by a participant to discount the rewards [3]:

*V* = *A*/(1 + *kD)*,

where *V* = subjective value of the delayed reward (POI), *A* = full amount of the delayed reward, *k* = empirically determined constant proportional to the degree of delay discounting (i.e., discounting function), and *D* = delay duration. According to this equation, the larger the discounting function (*k*) the individual applies, the more rapidly the value of the reward decreases based on its delay in time, theoretically reflecting greater impulsivity. Nonlinear regression was used to fit the preceding equation and the *R^2^* values were used to determine individual model fits. Erratic subjects and those with *R^2^* values below 0.30 were excluded from principal analyses [4]. Administration of the DDT took approximately 20 minutes.

Of the total sample of 195 subjects, 8 were clearly uncooperative on the DDT and provided erratic responses, resulting in exclusion from subsequent analysis. Of the remaining 187 subjects, Mazur’s [3] equation provided a good fit overall, typically accounting for over 90% of the variance (median *R^2^* = 0.904, interquartile range [IQR] = .72 - .96), which is comparable to past studies [5, 6]. However, an additional 21 subjects did not meet the model fit criterion (*R^2^* < .30), and were excluded from consideration in the primary DDT analyses. This further increased the median *R^2^* value for the remaining 166 subjects (*R^2^* = .92, IQR = .82 - .97). As anticipated, subjects’ performance on the DDT topographically resulted in hyperbolic discounting curves, exhibiting precipitous initial discounting followed by more modest decreases based on delay. The fit to Mazur’s equation did not differ by genotype (Factorial ANOVA 2 (A1+/A1-) X 2 (L+/L-); results not shown). While a factorial ANOVA including season of birth and gender in addition to DRD2 and DRD4 also showed no bias (not shown), one that only included season of birth in addition to genotypes did show bias. This included an association between DRD2 A1+ genotypes and poorer model fits (mean A1+=.553, A1-=.7873; F[1,179]=4.167, p=.043) and an interaction between DRD2 and season of birth that probably drives the DRD2 primary effect such that A1+/not-winter borns have much lower model fits than the remaining groups (A1+/not-winter=.288, F[1,179]=4.405, p=.037).

1. Petry NM: **Delay discounting of money and alcohol in actively using alcoholics, currently abstinent alcoholics, and controls.** *Psychopharmacology* 2001, **154:**243-250.

2. Petry NM: **Discounting of delayed rewards in substance abusers: relationship to antisocial personality disorder.** *Psychopharmacology* 2002, **162:**425-432.

3. Mazur JE: **An adjusting procedure for studying delayed reinforcement.** In *Quantitative analyses of behavior, The effect of delay and of intervening events on reinforcement value.* *Volume* 5. Edited by Commons ML, Mazur JE, Nevin JA, Rachlin H. Hillsdale, NJ: Erlbaum; 1987: 55–73

4. Reynolds B, Schiffbauer R: **Measuring state changes in human delay discounting: an experiential discounting task.** *Behavioural Processes* 2004, **67:**343-356.

5. Green L, Myerson J: **A discounting framework for choice with delayed and probabilistic rewards.** *Psychological Bulletin* 2004, **130:**769-792.

6. Green L, Myerson J, Ostaszewski P: **Discounting of delayed rewards across the life span: age differences in individual discounting functions.** *Behavioural Processes* 1999, **46:**89-96.
