# Supplementary material for: Season of Birth and Dopamine Receptor Gene Associations with Impulsivity, Sensation Seeking and Reproductive Behaviors
Source: PLoS One. 2007 Nov 21;2(11):e1216. doi: 10.1371/journal.pone.0001216 (PMC2075470; doi:10.1371/journal.pone.0001216)
Supplement: File S3 — Genotype Protocols and Genotype and Allele Frequencies (0.07 MB DOC) [file pone.0001216.s003.doc]

***DRD2 TaqI A* and *DRD4 48bp VNTR* Genotyping Protocols**

***DRD2 TaqI A*:**

The PCR reaction contained 0.5 µM forward (5' CAC GGC TGG CCA AGT TGT CTA 3’), 0.5 µM reverse primers (5’ CACCTTCCTGAGTGTCATCAA3’), 200 µM dNTP, 2.5 mM MgCl_2_, 0.25 units HotStar Taq (Qiagen), 1X Buffer (Qiagen), 2.5 µl DNA template in a total volume of 10 µl. Cycle conditions were: 15 min denaturation at 95°C, 40 cycles of 30 s denaturation at 94°, 30 s annealing at 55°, 1 min extension at 72° and one final extension of 7 minutes at 72°. The PCR product was digested with *Taq*I enzyme (New England Biolabs) overnight at 65° and visualized under UV on a 1.4% ethidium bromide agarose gel with 100 bp ladder. The 300 bp PCR product is not cut by the restriction enzyme in A1 alleles, and A2 alleles yield a 125 and 175 bp fragment. *DRD2 TaqI A* was typed successfully in 100% of the subjects.

***DRD4 48bp VNTR*:**

The PCR reaction contained 1x Q-Solution (Qiagen), 1x Buffer (Qiagen), 1 µM Primer 1 (5’ GCGACTACGTGGTCTACTCG 3’), 1 µM Primer 2 (5’ AGGACCCTCATGGCCTTG 3’), 200 µM dATP, dTTP, dCTP and 100 µM dITP and dGTP, 0.3 units HotStar Taq (Qiagen), 1 µl of DNA template, in a total volume of 10 µl. Thermocycler conditions were: 15 min at 95° to activate the enzyme and denature the DNA, 40 cycles of 1 minute denaturation at 94°, 1 minute annealing at 55°, 1.5 minute extension at 72°, followed by one cycle of 10 minute extension at 72°. A 4R PCR product is 475 bp. Samples were visualized under UV on 1.4 – 2.0% ethidium bromide agarose gels with a 100 bp ladder. Some gels were placed in a 1x boric acid bath for 10-15 minutes to increase contrast and allow better visualization of 9R alleles. With different concentrations of DNA template we observed allelic dropout of both short and long alleles. Because of these problems of allelic dropout and a previous report of only 84% correspondence between *DRD4/48bp* genotype techniques [1], all samples were run at 1/1, 1/40 and 1/80 dilutions for confirmation. If a minor allele was consistently observed at any dilution, the subject was considered to have that allele. *DRD4 48bp* genotyping had a 99% success rate with one subject who yielded no PCR product and another whose results varied between runs.

### ***DRD2 TaqI A* allele frequencies, genotypes and genotype classifications**

A1+ subjects have at least one A1 allele and A1- subjects are homozygous for A2.

| **Allele/Genotype** | **n** | **%** |
| --- | --- | --- |
| Allele |  |  |
| A2 | 274 | 70.3 |
| A1 | 116 | 29.7 |
| Total | 390 | 100.0 |
| Genotype |  |  |
| A2/A2 | 96 | 49.2 |
| A2/A1 | 82 | 42.1 |
| A1/A1 | 17 | 8.7 |
| Total | 195 | 100.0 |
| Genotype Classification |  |  |
| A1+ | 99 | 50.8 |
| A1- | 96 | 49.2 |
| Total | 195 | 100.0 |

### ***DRD4 VNTR* allele frequencies, genotypes and Genotype Classifications**

L+ subjects have at least one allele 7 repeats or longer in size and L- subjects have both alleles shorter than 7 repeats.

| **Allele/Genotype** | **n** | **%** |
| --- | --- | --- |
| Allele |  |  |
| 2 | 39 | 10.1 |
| 3 | 15 | 3.9 |
| 4 | 265 | 68.7 |
| 5 | 1 | 0.3 |
| 7 | 64 | 16.6 |
| 9 | 2 | 0.5 |
| Total | 386 | 100.0 |
| Genotype |  |  |
| 2/2 | 3 | 1.6 |
| 2/3 | 1 | 0.5 |
| 2/4 | 30 | 15.5 |
| 2/7 | 2 | 1.0 |
| 3/3 | 1 | 0.5 |
| 3/4 | 10 | 5.2 |
| 3/7 | 2 | 1.0 |
| 4/4 | 85 | 44.0 |
| 4/5 | 1 | 0.5 |
| 4/7 | 52 | 26.9 |
| 4/9 | 2 | 1.0 |
| 7/7 | 4 | 2.1 |
| Total | 193 | 100.0 |
| Genotype Classification |  |  |
| L- | 131 | 67.9 |
| L+ | 62 | 32.1 |
| Total | 193 | 100.0 |

1. Hamarman S, Fossella J, Ulger C, Brimacombe M, Dermody J**: Dopamine receptor 4 (DRD4) 7-repeat allele predicts methylphenidate dose response in children with attention deficit hyperactivity disorder: A pharmacogenetic stud**y*. Journal of Child and Adolescent Psychopharmacolog*y 2004**, 1**4:564-574.
